# Lethal disruption of the bacterial gut community in Eastern subterranean termite caused by boric acid

**DOI:** 10.1093/jee/toae221

**Published:** 2024-10-14

**Authors:** Aaron R Ashbrook, Melbert Schwarz, Coby Schal, Aram Mikaelyan

**Affiliations:** Department of Entomology and Plant Pathology, North Carolina State University, Raleigh NC, USA; Department of Entomology, Louisiana State University, Baton Rouge LA, USA; Department of Entomology and Plant Pathology, North Carolina State University, Raleigh NC, USA; Department of Population Health and Pathobiology, North Carolina State University, Raleigh NC, USA; Department of Entomology and Plant Pathology, North Carolina State University, Raleigh NC, USA; Department of Entomology and Plant Pathology, North Carolina State University, Raleigh NC, USA

**Keywords:** boric acid, termite control, gut microbiome, dysbiosis, wood protectants

## Abstract

The Eastern subterranean termite, *Reticulitermes flavipes* (Kollar) (Blattodea: Rhinotermitidae), is a significant pest, causing extensive damage to structures that amount to substantial economic losses. Boric acid is widely used for wood preservation due to its stability and broad-spectrum insecticidal properties, yet its impact on termite gut microbiomes and the implications of such effects remain understudied. Our study evaluates the dose-dependent mortality of *R. flavipes* upon being provided boric acid treated filter papers and investigates the resulting dysbiosis within the termite gut microbiome. Consistent with reports from other insects, mortality increased in a dose-dependent manner, with the highest boric acid concentration (203.7 µg/cm^2^ of filter paper) significantly reducing termite survival. 16S rRNA gene sequencing of the gut bacterial microbiome revealed notable shifts in composition, indicating boric acid-induced dysbiosis. Aside from an overall decrease in bacterial diversity, the relative abundance of some symbionts essential for termite nutrition decreased in response to higher boric acid concentrations, while several opportunistic pathogens increased. Our findings extend the understanding of boric acid’s mode of action in termites, emphasizing its ability to significantly modulate the bacterial symbiont community, which can have dire effects on termite biology. Considering its ability to protect wood from further termite consumption, our study supports the continued use of boric acid and related compounds for termite-resistant treatments for wood.

## Introduction

The Eastern subterranean termite, *Reticulitermes flavipes* (Kollar) (Blattodea: Rhinotermitidae), is a major wood-destroying pest with a wide distribution in the United States and around the world (Dedeine et al. 2016, Eyer et al. 2021). Subterranean termites display cryptic nesting behaviors, which often allow their colonies to go undetected when causing damage to structural wood (Oi 2022). Globally, termites impose an astonishing economic burden, with damages estimated at approximately USD 40 billion annually, with 80% of the damage caused by subterranean species (Rust and Su 2012, Oi 2022). The ability of *R. flavipes* and other termite species to inflict significant damage on wooden structures is attributed largely to their intricate symbiotic associations with gut microbiome (Brune 2014), which include protozoa (Gile 2024), bacteria (Brune and Dietrich 2015), and archaea (Protasov et al. 2023). This complex digestive symbiosis enables termites to effectively break down lignocellulose in wood (Watanabe and Tokuda 2010), which increases the risk of collapse of wooden structures (Scharf 2020).

The gut microbiome of termites is a complex and dynamic system that is susceptible to disruption by antibiotics, as has been observed in *Zootermopsis nevadensis* Hagen (Blattodea: Archotermopsidae) and *R. flavipes* (Rosengaus et al. 2011). Force-feeding starch to *R. flavipes* can precipitate a dramatic shift in microbiome composition and functionality, chiefly characterized by the elimination of cellulolytic protozoa and their associated bacteria (Ikeda-Ohtsubo et al. 2010, Mikaelyan et al. 2017). This vulnerability creates the potential for utilizing disruptive agents that could even enhance the efficacy of pesticides.

Boric acid is an inexpensive, broad-spectrum insecticide that has been widely used to protect wood from pest damage and can cause mortality in termites (Schubert 2000, Gentz and Grace 2006). While the full scope and mode of its action remain to be fully elucidated, it has been demonstrated to negatively impact digestion and nutrient absorption in a broad range of insects (Cochran 1995, Klotz et al. 2002, Habes et al. 2006, Gwokyalya and Altuntaş 2019). Notably in termites such as *R. flavipes* and *Coptotermes formosanus* Shiraki (Blattodea: Rhinotermitidae), preliminary studies have demonstrated that boric acid eliminates gut protozoa (Kard 2001), impairing termite digestion, and reinforcing its reputation as a ‘stomach poison’ (Ebeling 1978). However, the specific changes in the termite gut microbiome induced by boric acid remain under-explored.

Recent research on cockroaches, which share a close phylogenetic relationship with termites and have similarly complex gut microbiomes, highlights the disruptive effects of boric acid on these microbial communities (Jiang et al. 2021, Yang et al. 2021). Inspired by these insights, our study seeks to examine how boric acid alters the bacterial gut microbiome composition of *R. flavipes*, particularly in relation to its lethal and sublethal impacts. We aim to bridge this gap by focusing on two objectives: (1) characterizing the mortality of *R. flavipes* in response to various boric acid concentrations through feeding experiments and (2) investigating whether boric acid-induced disruption increases the prevalence of opportunistic pathogens in the bacterial gut community of *R. flavipes*. By conducting experiments over a span of 14 days, where termite workers are fed filter papers treated with different boric acid concentrations, we recorded their mortality and observed resulting alterations in their microbiomes after 7 days of boric acid treatment.

## Materials and Methods

### Insects

A colony of *R. flavipes* was collected from a forested area in Raleigh, NC, for use in experiments. Several hundred termites were maintained in plastic containers and provided with moist soil (Nature’s Care Organic & Natural Potting Mix with Water Conserve, Miracle-Gro, Marysville, OH), and pine shims (0.8 cm × 3.5 cm × 20 cm). The container was humidified by spraying water every 3 to 5 days. Termite colonies were maintained in an environmental rearing room at 26°C, 50% RH, on a 12:12 (L:D) cycle. Using a single colony allowed us to control for genetic and environmental variability, ensuring more consistent and interpretable results regarding the effects of boric acid on the termite gut microbiome.

### Boric Acid Bioassays

To administer boric acid to termites, filter paper discs (10 mm Whatman No 1, Cytvia, Marlborough, MA, USA) were initially stapled to plastic microscope coverslips (22 × 22 mm). Securing the treated filter papers to coverslips served to minimize direct contact between filter paper and the moist substrate, and prevent loss of boric acid to the substrate through leeching. This separation also allowed us to accurately weigh the papers by reducing particulate contamination from the substrate. These papers were subsequently treated with either 4 µl of distilled water (control) or 4 µl of boric acid solutions at various concentrations (0.125%, 0.25%, 0.5%, 1%, and 4% boric acid). This range of solutions resulted in the corresponding amounts of 5, 10, 20, 40, and 160 µg of boric acid per paper, or 6.4, 12.7, 25.5, 50.9, and 203.7 µg of boric acid per cm^2^ of filter paper, respectively. Following treatment, the papers were air-dried for 24 h in a convection oven (BOF-102, Being Scientific, Ontario, CA, USA) maintained at 32°C.

Prior to use in experiments, the filter papers, along with the plastic covers slips, were weighed using a precision balance (Explorer EX224, Ohaus, Wood Dale, IL, USA) to estimate the amount of paper consumed by the termites. The initial weight averaged 226 mg ± 0.26 mg (SD, *n* = 18). After the assay, the filter papers were dried (for 24 h at 32°C), and reweighed with the final weight averaging 222 ± 0.3 mg. The average weight of the filter papers alone was 6.8 mg. The amount of paper consumed during the bioassay was determined by subtracting the final weight from the initial weight, and this difference was used for statistical analysis.

For experiments, worker termites were removed from the colony using an aspirator. Groups of 10 worker termites were then placed in plastic Petri dishes (100 × 10 mm) containing 15 ml of sand moistened with 5 ml of tap water. All termites were starved for 24 h prior to the bioassay. Termites were then offered one filter paper treated with water (control treatment) or with a specific concentration of boric acid (3 replicates per treatment) in a no-choice assay. Mortality checks were conducted every 24 h over 14 days. Termites were deemed to be dead if they did not exhibit any movement in response to gentle prodding with feather-tip forceps. Dead termites were removed from the Petri dish to prevent necrophagy, which could potentially confound the results pertaining to direct consumption of boric acid. Bioassays were conducted in incubators at 26°C, 90% RH, in continuous darkness. Termites fed untreated filter papers served as controls, and each treatment was replicated three times.

### Analysis of Termite Survivorship and Consumption of Boric Acid-Treated Papers

Mortality data for termites fed different concentrations of boric acid during the 14-day experimental period were analyzed using Kaplan–Meier survival analysis in SPSS 27 (IBM, Armonk, NY, USA). Different concentrations and controls were compared in a pairwise manner using a log-rank test to determine statistical differences in survival times. Insects that survived beyond the 14-day period were right censored (i.e., their exact time of death was not identified, but they were known to have lived at least 14 days). To determine the hazard ratios for different treatments, a single proportional Cox hazard regression was conducted. The water controls in each experiment were used as the baseline of comparison of hazard regression and statistical separation of different treatments. When possible, mean survival times (MSTs), median survival times, and relative log hazard ratios were estimated for the Kaplan–Meier survival analysis and Cox hazard regression. The remaining weight of the treated filter papers was used to test for statistical differences between the amounts consumed in different treatments using analysis of variance (ANOVA) and Dunnett’s method for means comparison with individual treatments to the control group (untreated filter papers) (JMP 17, Cary, NC, USA).

### Gut Sampling, DNA Extraction, and Sequencing

To investigate the impact of boric acid on the gut microbiome of *R. flavipes*, a separate set of termites from the same colony was utilized, following a setup similar to the previously described mortality assay. Termites were isolated using an aspirator and placed in Petri dishes. Twenty worker termites per Petri dish were starved for 24 h, and then provided 2 filter papers treated with either water or a specific concentration of boric acid, as described earlier. The use of 20 termites (compared to 10 in the mortality assays) and 2 filter papers ensured that sufficient live termites were available for microbiome sequencing. Mortality was monitored every 24 h for 7 days and any dead individuals were promptly removed to prevent necrophagy. Control treatments were filter papers treated with water and termites provided with no paper. Each treatment was replicated three times.

On the seventh day of the experiment, 10 worker termites from each replicate were anesthetized on ice packs for dissection (The sampling time point of day 7 was chosen based on the median survival time in the 160 µg boric acid treatment). The gut was carefully removed by restraining the head with forceps and gently pulling the terminal segments of the abdomen with another forceps. Each termite gut was then placed in ZR BashingBead lysis tubes (Zymo Research, Irvine, CA, USA), suspended in 1 ml of ZR BashingBead buffer, and homogenized using a Benchmark Scientific D2400 Bead Beater (Sayreville, NJ, USA) for 4 cycles at 6.0 m/s for 11 s followed by 30 s of rest between each cycle. The homogenized gut samples were then stored at −80°C until DNA extraction. Each treatment and control group were replicated three times.

DNA from the homogenized termite guts was extracted using the Quick-DNA Fecal/Soil Microbe Microprep Kit (Zymo Research) following kit instructions. The bacterial community in the dissected guts of *R*. *flavipes* from control and boric acid-treated groups was characterized via amplicon sequencing of the 16S rRNA gene. Polymerase chain reaction (PCR) primers S-D-Bact-0341-b-S-17 (5ʹ-CCTACGGGNGGCWGCAG-3ʹ) and S-D-Bact-0785-a-A-21 (5ʹ-GACTACHVGGGTATCTAATCC-3; Klindworth et al. 2013), each with a unique 8-bp-barcode at the 5ʹ end, were utilized to amplify the V3–V4 region of the 16S rRNA gene. PCR was performed with 50 μl reactions prepared with 10–50 ng of genomic DNA, 0.4 μM forward and reverse primers, 25 μl Taq polymerase master mix red (PCR Biosystems, Wayne, PA, USA), and 20 μl molecular-grade water. The following PCR program was used: 30 s of denaturation at 95 °C, followed by 25 cycles of 20 s at 95 °C, 20 s at 58 °C, 30 s at 72 °C, and a final elongation step at 72 °C for 3 min. Amplicons were then cleaned using the DNA Clean and Concentrator-5 Kit (Zymo Research) following the manufacturer’s instructions. Purified amplicons were quantified and commercially sequenced at Novogene (Beijing, China) using the Illumina MiSeq platform.

### Microbiome Analysis

Processing and analysis of sequence data were conducted in line with the methodologies outlined by Schwarz et al. (2023), utilizing *mothur* (Schloss et al. 2009) for sequence processing and stringent quality control measures, followed by chimera removal and taxonomic classification. Briefly, we excluded contigs displaying ambiguities or homopolymer regions exceeding 10 bases. Additionally, sequences shorter than 200 bases, or those with an average quality score below 25, or a window average of 25 (over a window size of 50), were also eliminated. The remaining sequences were then aligned against the SILVA group’s comprehensive 50,000-position small subunit rRNA gene alignment. After the removal of chimeric sequences, the high-quality sequences were classified using the RDP Naïve Bayesian Classifier (Wang et al. 2007) implemented in *mothur*, referencing the SILVA database for taxonomic information (Quast et al. 2013). Following classification, sequences identified as originating from chloroplast or mitochondria were excluded from the subsequent ecological analyses.

To compare the alpha diversity of the termite microbiome across varying concentrations of boric acid, we calculated the Shannon diversity index using the *vegan* package (Dixon 2003) in R (R Core Team 2021) on the taxonomic composition of samples at the genus level. ANOVA was then conducted to test for significant differences in alpha diversity among the treatment groups. Beta diversity among the bacterial communities was assessed at the genus level using the Morisita-Horn metric, which assesses differences in community structure among samples, via the vegan package. The resulting distance matrix was analyzed through two-dimensional non-metric multidimensional scaling (NMDS; using the metaMDS function in *vegan*) to visualize dissimilarity in gut community structure across treatments. Additionally, permutational ANOVA (PERMANOVA) was performed on the Morisita-Horn distances using the adonis function in vegan to statistically evaluate the differences in termite gut microbiome composition among termite groups exposed to varying concentrations of boric acid.

A simple linear regression was conducted for each bacterial phylum in the dataset using the core lm() function in R to identify differences in their abundance relative to boric acid concentration. In this analysis, the relationship between bacterial abundance (response variable) and boric acid concentration (predictor variable) was modeled as Abundance = β_0_ + β_1_ × concentration + ε, where β_1_ represents the change in abundance per unit change in concentration. To explore differences at a finer taxonomic resolution, a random forest model was employed at the genus level using the *randomForest* package (Liaw et al. 2002) in R. This model ranked the bacterial genera by their importance in contributing to the observed differences in the termite gut microbiome under varying boric acid treatments. The model, built using genus-level abundance data, ranked the taxa based on their importance measured by the mean decrease in Gini Index, ensuring the identification of the most influential genera. The log-transformed relative abundance values of the top 30 taxa were then visualized using a heatmap (*pheatmap* package; Kolde and Kolde 2015), highlighting their distribution across different treatment groups and providing a clear representation of the taxa driving the observed differences in microbial community structure.

## Results

### Survival of *R. flavipes* After Feeding Upon Boric Acid-Treated Papers

**Fig. 1.**
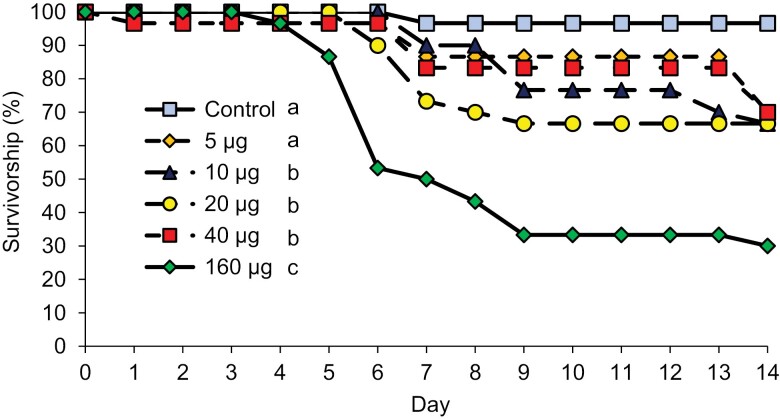
Mean proportional survival over time for *R. flavipes* individuals that were fed filter paper with water and varying amounts of boric acid (in µg). Each treatment represents 30 termites (3 replicates, each with 10 workers). Differences in the mean survival time determined by a log-rank test are represented by the letters in the figure legend. Treatments that do not share the same letter are significantly different (*P* < 0.05).

Survivorship of *R. flavipes* was significantly impacted by the concentration of boric acid in a dose-dependent manner (Fig. 1, Table 1), with different treatments significantly impacting termite survivorship (overall model, Chi-square = 48.1, df = 5, *P* < 0.005). In comparisons between treatments, we found no significant differences between the control and 5 µg of boric acid per paper, the lowest tested dose (Chi-square = 1.9, df = 1, *P* = 0.16). In the lower dose treatments, mortality began on day 6 or 7. Termites being fed filter papers treated with 10, 15, or 20 µg of boric acid exhibited similar mortality rates (Chi-square = 0.001 − 2.2, df = 1, *P* > 0.05), but they were significantly different from the lower dose as well as from the highest dose. The highest mortality was observed in the 160 µg boric acid treatment, which was significantly different from all other treatments (chi-square = 8.7 − 29.5, df = 1, *P* < 0.003). Some minimal mortality occurred after one day, but survivorship in the 160 µg boric acid treatment began to decline on day 4 and continued to decline until the end of the experiment. In the higher concentration treatments, mortality stabilized after day 9, suggesting that feeding had ceased. A similar pattern was observed in the lower concentration treatments (e.g., 5 and 10 µg), where a further decline in survival on days 12 and 13. As mentioned earlier, the median survival time in the 160 µg boric acid treatment was 7 days, which was therefore selected as the sampling point for the microbiome analysis in subsequent experiments.

**Table 1.** Kaplan–Meier estimates of mean survival time ± standard error for *R. flavipes* workers that were fed filter papers treated with different amounts of boric acid

Treatment	Mean survival time ± SE (days)	Median survival time (days)	Relative log hazard ratio (95% CI)^a^	Statistical significance relative to control^b^
Control	13.8 ± 0.3	≥14	–	a
5 µg	12.8 ± 0.6	≥14	4.2 (0.5 − 38.1)	a
10 µg	12.4 ± 0.5	≥14	9.7 (1.2 − 76.5)	b
20 µg	11.7 ± 0.6	≥14	11.8 (1.5 − 92.0)	b
40 µg	12.7 ± 0.6	≥14	9.8 (1.2 − 77.2)	b
160 µg	9.1 ± 0.7	7	34.2 (4.5 − 255)	c

Hazard ratios as determined by a Cox-Hazard regression are also reported.

^a^The relative log hazard ratio represents the logarithm of the hazard ratio, which quantifies the effect of boric acid concentration on termite mortality relative to the control group. A higher log hazard ratio indicates a greater risk of mortality associated with the treatment compared to the baseline (control), with the values adjusted for the logarithmic scale to allow easier comparison across different treatment levels. Ratios for treatments used as the baseline for comparisons are not reported.

^b^Treatment groups that do not share letters are significantly different (pairwise Chi-square tests, *P* < 0.05).

**Fig. 2.**
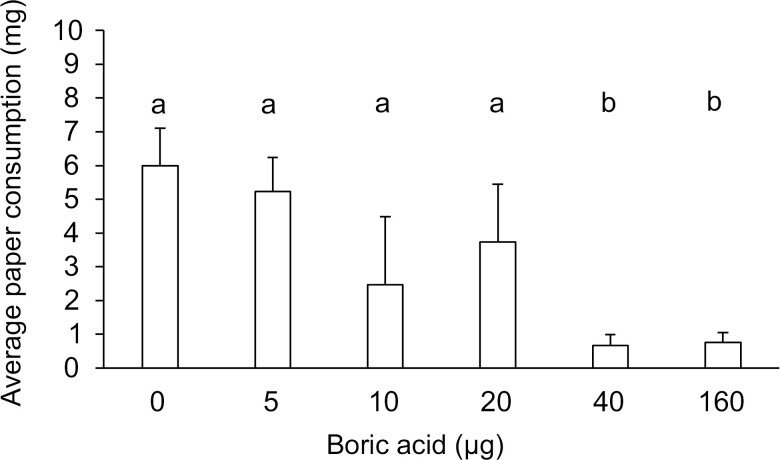
Average (± SEM) amount of paper consumed by *R. flavipes* based on treatment type. The 0 µg treatment served as the control group that was fed filter paper treated with water. Statistically significant differences in the average amount of paper consumed are indicated by different letters above the bars. Treatments not sharing the same letter are significantly different (ANOVA, Dunnett’s method, *P* < 0.05).

The amount of paper consumed varied depending on the boric acid concentration present (Fig. 2, ANOVA, df = 5, 17, *F* = 3.48, *P* = 0.0355). Fresh filter paper weighed 6.8 mg. Post hoc means comparisons via the Dunnett’s method revealed significant differences between treatments, with the control and the 5 µg treatment groups being statistically similar (*P* > 0.05), with all the paper being consumed in 2 replicates of the control and one replicate of the 5 µg treatment group. The 10 and 20 µg boric acid treatment groups had similar consumption to the control treatments as well, with an average consumption of 2.5 ± 2.0 and 3.7 ± 1.7 mg, respectively. Termites in the 40 and 160 µg treatment groups consumed the least amount of paper at an average 0.67 ± 0.3 and 0.77 ± 0.33 mg per treatment, respectively, and each was statistically different from control treatments (*P* = 0.038 and 0.035).

### Termite Gut Bacterial Community in Relation to Boric Acid Concentration

We assessed the ability of boric acid to alter the microbial community of termites by feeding them different concentrations which also significantly impacted their survivorship. Data processing in *mothur* yielded a total of 9.87 million sequences from the 18 samples, with 548,456 sequences on average obtained per sample. Seventy-nine percent of all sequences (from all samples) were classified to the genus level using the Silva (v138) non-redundant database. This high classification success allowed us to conduct all downstream ecological analyses at the bacterial genus level. ANOVA comparing alpha diversity across 2 boric acid concentration groups revealed a significant difference (*F* = 23.89, df = 1, 16, *P* < 0.005). The PERMANOVA results indicate a highly significant effect of boric acid concentration on the termite gut microbiome structure (*F* = 46, *R*^2^ = 0.7417, *P* = 0.001), which suggest that around 74% of the observed variation in community structure can be explained by the grouping variable, i.e., boric acid concentration.

**Fig. 3.**
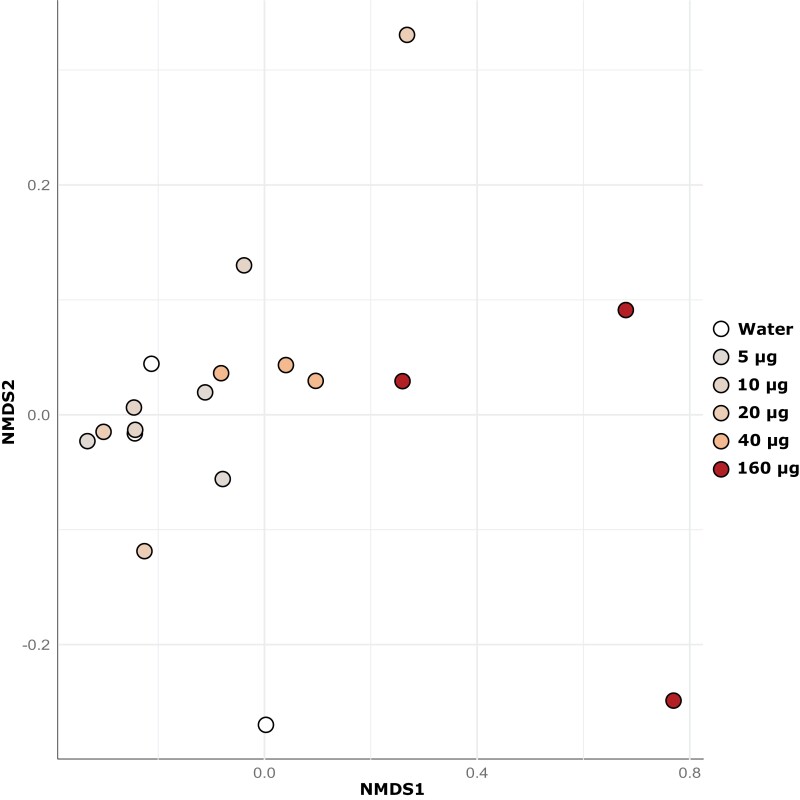
Non-metric multidimensional scaling (NMDS) plot depicting the structural dissimilarities (based on the Morisita-Horn metric) between termite gut bacterial communities in response to feeding upon filter papers treated with varying boric acid amounts. The two-dimensional solution had a low stress value (0.045), indicating a good representation of the data in reduced dimensions. Each point represents the compositional similarity of the termite gut communities, with closer points indicating more similar bacterial community compositions.

In the NMDS ordination plot (Fig. 3; Stress = 0.045) based on Morisita–Horn distances, a distinct separation is evident between the control group and the samples subjected to the highest boric acid dose (160 µg per filter paper). This separation underscores the significant disruption of bacterial community structure at this boric acid level, as further supported by the substantial proportion of explained variance in the PERMANOVA analysis. While there is a visible gradient across the boric acid doses, the absence of a distinct separation between the control group and the 40 µg boric acid treatment group suggests that lower concentrations of boric acid may only minimally affect the gut microbiome structure (as indicated by the overlapping points in the NMDS plot). This observation aligns with the PERMANOVA result, where the high *R*^2^ value indicates that higher concentrations of boric acid are driving the structural changes in the bacterial microbiome.

### Exposure to Boric Acid Causes Taxonomic Shifts in Termite Gut Community

**Fig. 4.**
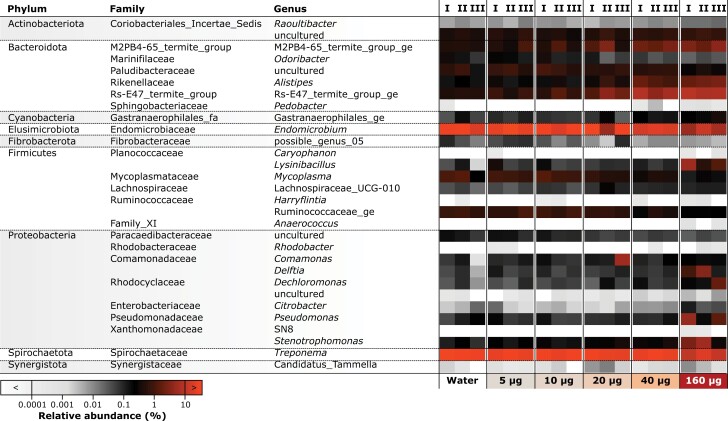
Heatmap depicting the distribution and abundance of the top 30 bacterial genera in the gut microbiome of *R. flavipes*, ranked by their contribution to group differentiation as determined by a random forest model. Each column represents a replicate of a treatment group (indicated below the heatmap), and variations in color intensity correspond to the log-transformed relative abundance of each genus, illustrating the impact of different concentrations of boric acid on bacterial community structure. For a more detailed exploration of changes in bacterial community structure, see Supplementary Table S1.

To uncover specific taxonomic shifts in the termite microbiome in response to the boric acid treatments, we used a Random Forest machine learning approach. We selected the top 30 genera that exhibited marked shifts in relative abundance across the gradient of boric acid treatments (visualized in the heatmap in Fig. 4). The heatmap reveals several patterns in the distribution and abundance of bacterial taxa in relation to boric acid exposure in termites (for a more comprehensive exploration of the changes in the gut microbiome at various taxonomic levels, see Supplementary Table S1). Notably, genera within the phylum Proteobacteria, including *Pseudomonas*, *Citrobacter*, and *Stenotrophomonas*, showed pronounced variations in relative abundance, particularly at the higher dose of 160 µg. Conversely, some members of the phylum Actinobacteriota, such as the ‘Coriobacteriales *incertae sedis*’ and the ‘uncultured’ group, displayed a decrease in relative abundance with increasing concentration of boric acid, with the lowest presence observed in the 160 µg treatment. Phyla such as the Fibrobacteraceae from the Fibrobacterota, which have been associated with cellulose degradation in the guts of higher termites, show a reduction in abundance with increasing boric acid.

Linear regression analysis further allowed us to interrogate the impact of increasing boric acid concentrations on the distribution and abundance of various bacterial taxa within the termite gut. It unveiled both significant positive and negative relationships across different bacterial lineages. Broad patterns in gut community structure reflecting boric acid concentrations were observed already at the phylum level. Bacteroidota and Firmicutes showed a strong positive relationship with increasing boric acid concentration, evidenced by their *P*-values (0.0102 and 0.0437, respectively) and *R*^2^ values (0.41 and 0.28, respectively). Conversely, Spirochaetota and Elusimicrobiota displayed significant negative trends, with *P*-values of 1.75e−05 and 0.0051, respectively. The high *R*^2^ values for Spirochaetota (0.77) and Elusimicrobiota (0.47) indicate that a significant portion of the variance in abundance for these phyla can be attributed to boric acid concentrations.

Diving deeper at the genus level, *Alistipes* (Phylum: Bacteroidota) demonstrated a significant positive relationship with boric acid concentration (*P* = 1.47e-05), with an *R*^2^ value of 0.78. The abundance of *Alistipes* increased from 0.46% ± 0.03% in water-treated samples to 2.97% ± 1.05% in samples treated with 160 µg of boric acid. *Raoultibacter* (Phylum: Actinomycetota), despite its significant shift (*P* = 3.23e−05, *R*^2^ = 0.75), remained a comparatively rare taxon, with a relative abundance of less than 0.05% across all treatments.

Significant positive trends were also identified for several other taxa, including ‘Rs-E47_termite_group_ge’ (Phylum: Bacteroidota), *Lysinibacillus* and *Mycoplasma* (Phylum: Firmicutes), as well as *Pseudomonas*, *Delftia*, *Dechloromonas*, and *Stenotrophomonas* (Phylum: Proteobacteria). These taxa demonstrated substantial increases in mean relative abundance—ranging from 18- to 45-fold—between control filter papers and those supplemented with 160 µg of boric acid. For instance, *Lysinibacillus* surged from 0.06% ± 0.07% in the control to 2.53% ± 2.76% in termites exposed to boric acid. Similarly, the abundance of Rs-E47_termite_group_ge escalated from 0.48% ± 0.04% to 8.43% ± 1.54%.

Only one of the genera shortlisted by our random forest approach, *Endomicrobium,* presented a significant negative trend (*P* = 0.00512, *R*^2^ = 0.47), indicating a drop in abundance with rising boric acid concentrations. Its relative abundance diminished from 26.38% ± 6.36% in the control group to 8.32% ± 4.39% in the 160 µg boric acid treatment group, highlighting a considerable impact of boric acid on this taxon’s presence within the termite gut.

## Discussion

Our study builds upon existing evidence to further validate boric acid’s lethal impact on termites, with survivorship in the eastern subterranean termite *R. flavipes* dropping in a dose-dependent manner (Fig. 1, Table 1). This finding supports previous observations of dose-dependence in *R. flavipes*(Su et al. 1994), and the termites *C. formosanus* (Su et al. 1994, Gentz and Grace 2009, Gentz et al. 2009) and *Heterotermes indicola* (Wasmann) (Blattodea: Rhinotermitidae) (Farid et al. 2015). The highest amount tested in our study was 160 µg of boric acid per 6.8 mg of filter paper, which corresponds to approximately 23,529 ppm (calculated as 160 µg/6,800 µg to convert to ppm). This concentration led to significant termite mortality, which parallels the mortality observed at lower concentrations (10,000 ppm) reported by Farid et al. (2015). This dose-dependent effect has been similarly documented for boric acid when ingested by *Blattella germanica* L. (Blattodea: Ectobiidae) (Cochran 1995, Habes et al. 2001, 2006, Gore and Schal 2004, Jiang et al. 2021), *Galleria mellonella* L. (Lepidoptera: Pyralidae) (Gwokyalya and Altuntaş 2019), *Apis mellifera* L. (Hymenoptera: Apidae) (da Silva Cruz et al. 2010), and *Cimex lectularius* L. (Hemiptera: Cimicidae) (Sierras et al. 2018).

We found that termites consumed less paper in a dose-dependent manner, with the least amount consumed from papers treated with the highest concentrations of boric acid (Fig. 2). Although boric acid does not cause an immediate repellent effect, our results and those of Farid et al. (2015) show that it ultimately leads to reduced feeding. Other studies have shown that *R. flavipes*, *C. formosanus* and *Coptotermes gestroi* (Wasmann) (Blattodea: Rhinotermitidae), consume less wood when treated with higher concentrations boric acid solutions (Kard 2001, Casarin et al. 2009). While it might be speculated that reduced feeding is a result of termite mortality, our data suggest otherwise. The 40 µg treatment, which had lower mortality than the 160 µg treatment, still resulted in similar levels of paper consumption, indicating that sublethal effects beyond mortality likely contribute to the observed reduction in feeding. It is important to note that this study utilized a no-choice assay, which may not fully capture potential avoidance behaviors or feeding deterrence. Future research incorporating choice assays could provide a more nuanced understanding of these effects.

While the specific mode(s) of action of boric acid in termites remains underexplored, evidence from prior studies with termites and several other insect species indicates its significant impact on the digestive system. Studies, including Cochran (1995) and Habes et al. (2006), have demonstrated that boric acid induces structural changes in the midgut of *B. germanica*, potentially impairing nutrient absorption. It was later shown by Yang et al. (2021) that boric acid can create pores in the midgut lining of *B. germanica*, facilitating infection by *Metarhizium anisopliae* (Metchnikoff) (Hypocreales: Clavicipitaceae) spores. Given that cockroaches and termites are close phylogenetic relatives, it is plausible that termites experience similar digestive disruptions. However, the potential impairment of the midgut has also been observed in Argentine ant *Linepithema humile* (Mayr) (Hymenoptera: Formicidae) (Klotz et al. 2002) and the honey bee *A. mellifera* (da Silva Cruz et al. 2010), underscoring the disruption of digestive physiology as an important contributor to the insecticidal effects of boric acid.

Our results reveal that boric acid induces significant alterations in alpha diversity and structural changes within the termite gut microbiome (Fig. 3). This aligns with observations by Jiang et al. (2021), where boric acid was shown to disrupt the normal gut microbiome composition in *B. germanica*, suggesting a similar mechanism might be at play in termites. The decrease in alpha diversity and significant shifts in microbial community composition, especially at higher boric acid concentrations, are indicative of dysbiosis, defined as an imbalance or shift in a naturally present microbiome of a healthy host (Petersen and Round 2014). Given the reliance of termites on their gut microbiome for the symbiotic digestion of wood, such an imbalance seems to magnify the detrimental effects of boric acid.

A loss of diversity in the gut microbiome is typically tied to reduced functionality, and is often accompanied by a loss of beneficial symbionts and an expansion of opportunistic pathogens (Petersen and Round 2014). The clear clustering and separation observed in the NMDS plot for termites exposed to filter paper treated with 160 µg of boric acid compared to the control group illustrates the significant impact of boric acid on gut community structure (Fig. 3). This pattern not only confirms the presence of dysbiosis but also suggests that higher concentrations of boric acid may lead to more pronounced shifts in microbial populations, potentially disrupting critical processes such as digestion, nutrient absorption, and pathogen resistance within the termite gut. However, the noticeable dispersal in clustering among replicates treated with higher concentrations of boric acid (Fig. 3) indicates variability in the response of the termite gut microbiome to the treatment. This variability, coupled with the observed shifts in the relative abundance of certain bacterial genera (Fig. 4), suggests that the dysbiotic effects of boric acid on the termite gut microbiome are not deterministic and may vary depending on a complex range of factors.

Our screening for microbial taxa contributing to the overall differences observed in microbiome structure revealed a downward trend (with increasing boric acid exposure) in the abundance of key genera, such as *Endomicrobium* (Fig. 4) that are major endosymbionts of *Trichonympha* flagellates. The drop in the relative abundance of these flagellate endosymbionts reinforces the findings of Kard (2001), who found boric acid at 4% solution (equivalent to our 160 µg treatment) to be effective at disrupting the flagellate community of *R. flavipes*. Other forms of defaunation (experimental removal of flagellates) by force-feeding starch or other treatments have also been shown to markedly reduce the abundance of this bacterial endosymbiont (Ikeda-Ohtsubo et al. 2010, Mikaelyan et al. 2017). Despite the drop in abundance, the persistence of *Endomicrobium* in boric acid-treated termites may represent lineages of ‘putatively free-living endomicrobia’ observed in multiple species of starved or defaunated lower termites (Ikeda-Ohtsubo et al. 2010, Mikaelyan et al. 2017). However, short-read 16S sequencing cannot provide the phylogenetic resolution necessary to conclusively distinguish between endosymbionts and free-living lineages (Mikaelyan et al. 2017).

The observed negative relationship between boric acid concentration and the Spirochaetota phylum, essential for providing vital microbial services (Tokuda 2021), such as lignocellulose degradation (Mikaelyan et al. 2014, Tokuda et al. 2018), nitrogen fixation (Lilburn et al. 2001, Ohkuma et al. 2015), and homoacetogenesis (Leadbetter et al. 1999, Ottesen and Leadbetter 2011) highlights that its depletion poses a significant detriment to termite metabolism.

At the same time, the observed increase in certain genera, notably *Lysinibacillus*, *Pseudomonas*, and *Mycoplasma*, suggests a proliferation of opportunistic pathogens. *Lysinibacillus sphaericus* (previously known as *Bacillus sphaericus*) is recognized for its mosquito larvicidal properties (Chalegre et al. 2015, Rezende et al. 2019) and is closely related to several insect-pathogenic species (Nakamura 2000). The genus *Pseudomonas* similarly includes many pathogens of arthropods (Roobakkumar et al. 2011, Carrau et al. 2021, Hamze et al. 2022) with preadaptations to infect insects as specialists or as opportunists. When *Mastotermes darwiniensis* Froggatt (Blattodea: Mastotermitidae) is in a state of dysbiosis due to starch feeding, there is an increase in bacteria in their hindguts, that may be opportunists (Veivers et al. 1983). In *B. germanica*, boric acid exposure led to dysbiosis, characterized by a decrease in beneficial bacteria like *Bacteroides* and *Enterococcus*, and an uptick in potentially pathogenic *Weissella* species (Jiang et al. 2021). Similarly, when *Drosophila melanogaster* Meigen (Diptera: Drosophilidae) are in a state of dysbiosis, opportunistic bacteria in their guts, such as *Gluconobacter mortifer*, become pathogens (Lee and Lee 2014).

The dysbiosis observed in our study underscores the significant impact of boric acid on the termite gut microbiome, which could have important implications for termite health and survival in boric acid-treated environments. However, in addition to substantially altering gut microbiome composition in termites, boric acid could also potentiate the vulnerability of termites to external entomopathogens. As demonstrated by Yang et al. (2021), boric acid enhanced the virulence of *M. anisopliae* against cockroach *Blatella germanica* by altering the gut microbiome, and a similar mechanism could contribute to the overall effectiveness of boric acid as a termite management tool. Judging the extent of the impact of boric acid at the colony level and the role that trophallaxis could play in mitigating its effects in larger groups of termites would have to be addressed in future studies.

The decrease in alpha diversity and significant shifts in microbial community composition, especially at higher concentrations of boric acid, indicate a disruption of the gut microbiome’s equilibrium. This dysbiosis could further compromise termite health and resilience, beyond the direct toxicological effects of boric acid. Considering the substantial alterations in the gut microbiome and the increased termite mortality associated with boric acid consumption, our findings support the exploration of synergistic approaches for termite management. Our study contributes to the growing body of evidence on the utility of boric acid in termite management, not only as a direct toxicant but also as a disruptor of gut microbiome homeostasis. Future research should focus on elucidating the specific mechanisms by which boric acid induces dysbiosis and exploring its synergistic potential with microbial control agents, paving the way for innovative and sustainable termite control strategies.

## Supplementary data

Supplementary data are available at *Journal of Economic Entomology* online.

toae221_suppl_Supplementary_Table_S1

## Data Availability

The 16S rRNA gene sequence reads analyzed as part of this study are available in the NCBI Sequence Read Archive (SRA) repository under Bioproject ID PRJNA1096953.
